# Editorial: The role of gut microbiota-gut-brain axis in inflammatory bowel disease

**DOI:** 10.3389/fmed.2025.1569664

**Published:** 2025-03-03

**Authors:** Jiayin Yao

**Affiliations:** The Sixth Affiliated Hospital of Sun Yat-sen University, Guangzhou, China

**Keywords:** inflammatory bowel disease, microbiota-gut-brain axis (MGB axis), gut-brain axis, editorial, Crohn's disease

## Introduction

Inflammatory Bowel Disease (IBD), which includes Crohn's disease and ulcerative colitis, is a complex chronic inflammatory condition characterized by relapsing and remitting inflammation of the gastrointestinal tract. The pathogenesis of IBD is multifactorial and involves intricate interactions between genetic predisposition, immune dysregulation, environmental triggers, and microbial dysbiosis. In recent years, the gut microbiota-brain axis has emerged as a pivotal area of research, shedding light on the bidirectional communication between the gut microbiota, the enteric nervous system, and the central nervous system. This axis not only underscores the role of microbial communities in modulating host immunity but also highlights the profound influence of gut-brain interactions on the development, progression, and symptomatology of IBD.

This Research Topic focuses on the critical role of the gut microbiota-gut-brain axis in IBD, presenting a collection of four high-quality research articles that include comprehensive reviews and original studies. These contributions provide significant insights into the latest advancements in this field, offering novel perspectives on the mechanisms underlying IBD and potential therapeutic strategies.

## Review articles: synthesizing current evidence

Two review articles in this Research Topic systematically summarize the current state of research. The first, titled “*Targeting gut microbiota dysbiosis in inflammatory bowel disease: a systematic review of current evidence,”* provides a comprehensive evaluation of therapeutic strategies aimed at modulating gut microbiota dysbiosis (Farah et al.). The review emphasizes the potential of probiotics, prebiotics, fecal microbiota transplantation (FMT), and dietary interventions in restoring microbial balance and improving clinical outcomes in IBD patients. Notably, the authors highlighted the promising results of FMT in inducing remission in ulcerative colitis, as supported by recent clinical trials (1).

The second review, “*Exploring the role of IL-1*β *in inflammatory bowel disease pathogenesis,”* delves into the role of the pro-inflammatory cytokine IL-1β in driving intestinal inflammation (Aggeletopoulou et al.). The authors discussed how IL-1β contributes to mucosal barrier dysfunction, immune cell activation, and fibrosis in IBD. They also explored the potential of IL-1β-targeted therapies, such as anakinra and canakinumab, in mitigating disease severity. This review builds on previous findings that IL-1β signaling is a key mediator of inflammation in both Crohn's disease and ulcerative colitis (2).

## Original research: unveiling novel mechanisms

The original research articles in this Research Topic offer groundbreaking insights into the metabolic and immunological aspects of the gut microbiota-gut-brain axis in IBD. The first study, “*Alterations in tryptophan metabolism and de novo NAD*+ *biosynthesis within the microbiota-gut-brain axis in chronic intestinal inflammation,”* investigates the metabolic changes associated with chronic intestinal inflammation (Devereaux et al.). The authors revealed significant alterations in tryptophan metabolism and NAD+ biosynthesis pathways that are critical for maintaining intestinal homeostasis. These findings suggest that targeting these metabolic pathways may offer new therapeutic avenues for the treatment of IBD. This study aligns with previous research demonstrating the role of tryptophan metabolites, such as kynurenine, in regulating immune responses and gut-brain communication (3).

The second original study, “*The association of three vaccination doses with reduced gastrointestinal symptoms after severe acute respiratory syndrome coronavirus 2 infections in patients with inflammatory bowel disease,”* examines the impact of COVID-19 vaccination on gastrointestinal symptoms in IBD patients (Hong et al.). The findings indicate that three doses of mRNA vaccines are associated with a significant reduction in post-infection gastrointestinal symptoms, underscoring the importance of vaccination in this vulnerable population. This study adds to the growing body of evidence supporting the safety and efficacy of COVID-19 vaccines in IBD patients (4).

## Future directions and clinical implications

The studies featured in this Research Topic not only deepen our understanding of the gut microbiota-gut-brain axis in IBD but also pave the way for future research and therapeutic innovations. For instance, the exploration of microbial metabolites, such as short-chain fatty acids (SCFAs) and secondary bile acids, holds promise for the development of targeted therapies to restore gut-brain axis homeostasis. Additionally, the integration of multi-omics approaches, such as metagenomics, metabolomics, and transcriptomics, may provide a more comprehensive understanding of the complex interactions underlying IBD pathogenesis.

Moreover, the potential of psychobiotics—live microorganisms with mental health benefits—in modulating the gut-brain axis offers an exciting avenue for addressing the psychological comorbidities often associated with IBD, such as anxiety and depression. Recent studies have demonstrated the efficacy of certain probiotic strains, such as *Lactobacillus* and *Bifidobacterium*, in alleviating both gastrointestinal and neuropsychiatric symptoms in IBD patients (5).

## Conclusion

In conclusion, the gut microbiota-gut-brain axis represents a promising frontier in IBD research, offering novel insights into disease pathogenesis and potential therapeutic targets. The studies presented in this Research Topic underscore the importance of interdisciplinary approaches in unraveling the complexities of IBD and developing personalized treatment strategies. As research in this field continues to evolve, we anticipate significant advancements in precision medicine and improved quality of life for IBD patients.

Finally, we extend our gratitude to all the authors, reviewers, and readers for their invaluable contributions to this Research Topic. We hope that these findings will inspire further research and foster collaboration among scientists, clinicians, and patients to advance our understanding and management of IBD.
